# Scientific Scraps

**Published:** 1881-09

**Authors:** 


### SCIENTIFIC SCRAPS.

Between 600 and 700 different forms have been distinguished in snow crystals.

Pure water may be obtained from that which is impure, or from brine, by distillation.

Articles of food which would soon decay if exposed to the air may be long preserved in a vacuum.

In Switzerland the temperature of the bottom of deep, snow-fed lakes remains uniform during the year.

Respiration is slow combustion, in which carbon and other ingredients of the blood combine with oxygen.

Why will grass not grow under our trees? M. Paul Bert has shown that green light hinders the development of plants. Plants inclosed in a green glass frame wither and die as though they were in darkness. M. Regnard finds that plants specially require the red rays. If sunlight is deprived of the red rays the plants soon cease to thrive.

NIAGARA FALLS is so brilliantly illuminated by the electric light every evening that after paying the hackman you can easily see whether there is anything left in your pocketbook.—*Philadelphia News.*

